# Clinical observation on the treatment of displaced distal radial and ulnar fractures in children by closed manipulation combined with splinting

**DOI:** 10.3389/fsurg.2023.1199437

**Published:** 2023-09-19

**Authors:** Guangwei Wang, Liwei Huo, Yidong Xu, Gerong Dai, Zhong Yang, Jianwei Hu, Weinian Liu

**Affiliations:** ^1^ Department of Orthopaedics, The Third Affiliated Hospital of Guangzhou University of Chinese Medicine, Guangzhou, China; ^2^ Department of Orthopaedics, Guangzhou Orthopedic Hospital, Guangzhou, China

**Keywords:** distal radius and ulna, displaced fracture, children, closed manipulative reduction, splint fixation

## Abstract

**Objective:**

The aim of this study was to investigate the clinical efficacy of closed manipulation combined with splinting in the treatment of displaced distal radial and ulnar fractures in children.

**Methods:**

A total of 82 children with displaced fractures of the distal radial and ulnar segment who met the inclusion criteria and were treated as outpatients or inpatients in the orthopedic department of Guangzhou Orthopedic Hospital, from January 2016 to June 2022 were randomly divided into an observation group and a control group: 41 children in the observation group were treated with closed manipulation combined with splint fixation; 41 children in the control group were fixed with incisional repositioning elastic nails combined with internal plates. The Anderson efficacy grading, visual analog scale (VAS) score, fracture healing time, treatment cost, hospital days, and complications were observed and compared between the two groups.

**Result:**

The efficacy was evaluated according to the Anderson forearm fracture efficacy evaluation criteria, and the results of statistical analysis showed no statistically significant differences between the two groups (*P *> 0.05). At 3 and 7 weeks after treatment, the VAS scores of children in both groups decreased (*P *< 0.05), and the VAS scores in the observation group were significantly lower than those in the control group (*P *< 0.05), indicating that the observation group had a significant advantage in the relief of pain after treatment. The fractures healed in both groups after treatment with the two different methods, and the difference in healing time between the two groups was not statistically significant (*P* > 0.05). The length of hospital stay, treatment cost, and complication ratio were significantly lower in the observation group than in the control group (*P* < 0.05).

**Conclusion:**

In children with displaced fractures of the distal radial and ulnar segments, treatment by manual repositioning with external splinting can achieve satisfactory results with simple operation, low cost, short hospital stay, and few complications, which is especially suitable to be promoted in primary hospitals and has good social benefits.

## 1. Introduction

Fractures of the distal radius and ulna, mostly in children aged 6–12 years and with regular displacement, are distal to dorsal displacement of the radius, with overlapping displacement, and mostly green branch fractures of the ulna with little displacement, which also belong to the classical fractures specific to children (1, 2). The mechanism of injury and displacement is that the child falls with the forearm rotated forward and the palm of the hand landing first, with the wrist mostly landing in an extended dorsal extension position. During the fall, the downward gravitational force of the body and the upward reaction force of the ground are concentrated on the distal radial and ulnar segment, resulting in a fracture of the distal radial and ulnar segment, and due to the relative fixation of the distal end, the proximal end is displaced by body gravity to the palmar side of the distal fracture, forming a dorsal displacement of the distal fracture (3, 4). In children with displaced fractures of the distal radius and ulna, the fracture, if not treated correctly and effectively, can easily lead to wrist and forearm dysfunction, resulting in deformity of the injured arm, which can seriously affect the growth and development of the children (5).

The main goal of treatment of fractures of the distal radius and ulna in children is to restore the normal anatomy of the fracture site and improve the motor function of the forearm (6). There is no uniform treatment for fractures of distal radius and ulna in children, and although surgical treatment can achieve good repositioning results, there are surgical risks and the need for reoperation to remove the internal fixation; non-surgical treatment is less invasive, but it is not easy to maintain the repositioning results (7, 8). Huang et al. (9) concluded that patients with extremity fractures should be treated with manual repositioning, especially for patients with upper extremity fractures that do not damage the joint and are not severely separated, and that manual repositioning can significantly reduce injury and help fracture healing. However, Ali et al. (10) concluded that this treatment method, although less invasive, is not easy to maintain the therapeutic effect. In order to investigate the best treatment for displaced fractures of the distal radius and ulna in children, this study used incisional repositioning elastic nail combined with internal plate fixation and manual repositioning combined with splint fixation in children, respectively, and the clinical efficacy of the two treatments was observed.

## 2. Methods and patients

### 2.1. General information

A total of 82 children were included in the study, all of whom were treated in the orthopedic department of Guangzhou Orthopedic Hospital as outpatients or inpatients from January 2016 to June 2022. There were 66 boys and 16 girls, aged 5–14 years, with a median of 10 years; 46 cases were on the left side and 36 cases on the right side. Injury to treatment time was 1 h to 3 days, with a median of 1 day. The trial protocol was reviewed and approved by the Ethics Committee of Guangzhou Orthopedic Hospital [Ethical Code: Zhenggu (2023) Lunshen No. 202307].

### 2.2. Diagnostic criteria

According to the diagnostic criteria related to distal radius and ulna fractures (11), children have a clear history of trauma, pain or pressure, and local swelling at the site of injury; the child has a deformity or abnormal movement of the limb, bone rubbing sound, and functional impairment of the anterior wall; and the diagnosis of a double fracture of the lower segment of the ulnar radius is confirmed by computed radiography (CR) or radiography.

### 2.3. Inclusion criteria

The inclusion criteria were (1) closed injury; (2) meeting the above diagnostic criteria for double fractures of the ulnar radial trunk; (3) fracture of the middle and lower radioulna; (4) transverse shaped fracture with overlapping displacement of the fracture end; (5) aged 1–14 years; (6) fracture time <7 days; and (7) informed consent of the children or their guardian with a signed informed consent form.

### 2.4. Exclusion criteria

The exclusion criteria were (1) pathological fracture; (2) combined with craniocerebral injury or traumatic abdominal organ rupture and bleeding; (3) combined with other fractures; (4) combined with other diseases affecting the recovery of joint function; and (5) combined with serious medical diseases that cannot tolerate surgery and contraindicated for manual repositioning.

### 2.5. Treatment methods

A total of 82 children were divided into observation (*n* = 41) and control (*n* = 41) groups according to the random number table method.

**Figure 1:**
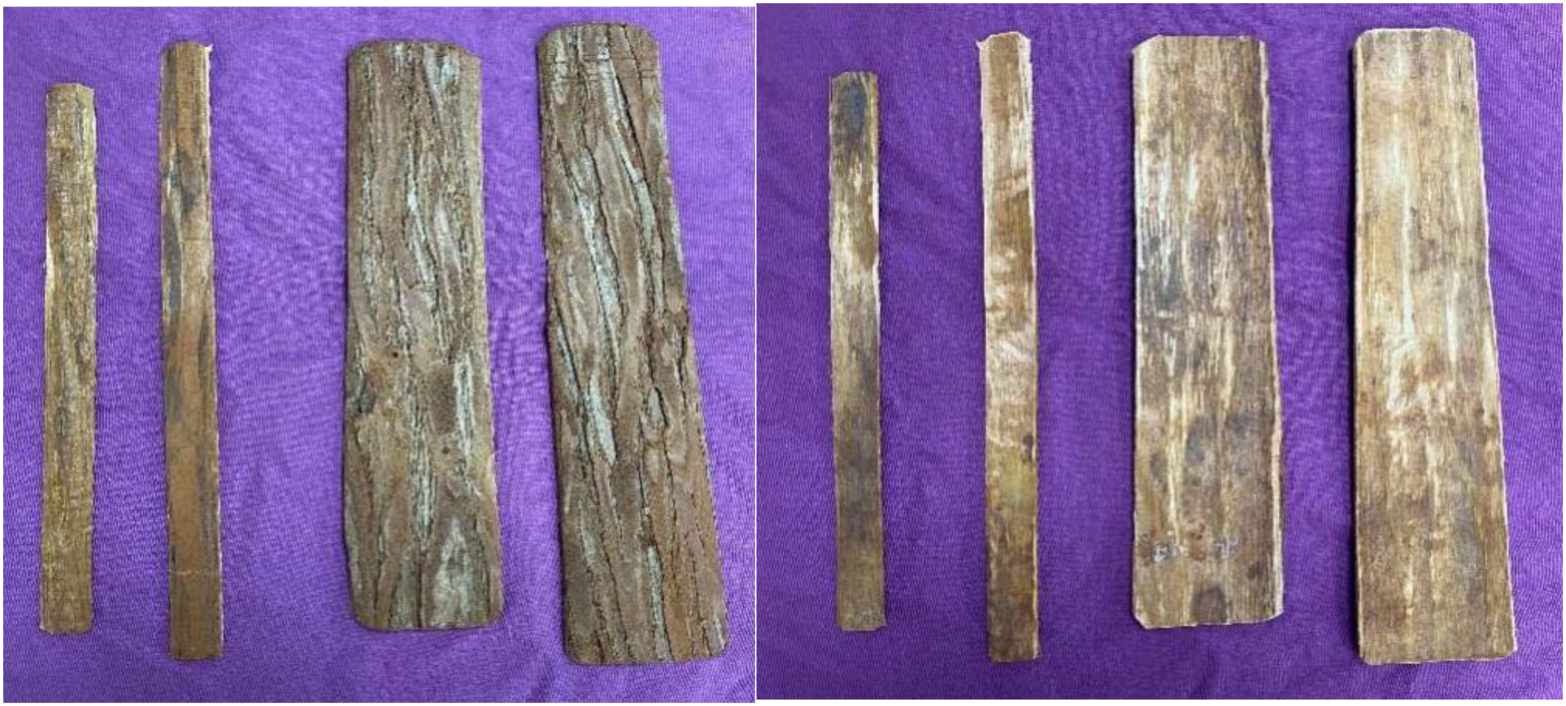
Splint.

Observation group: (1) Manual repositioning: The children in the observation group were placed in the supine position (the younger ones were carried into the arms by their parents) with the shoulders abducted 60°–90°, the elbows flexed 90°, and the forearms in the neutral position. First, two assistants continued antagonistic traction for 3–5 min, doing extraction and extension traction. In cases of angular deformity or minor shortening and displacement, repositioning by end lifting, squeezing and pressing, and bone splitting techniques could mostly be achieved. For larger overlapping displacements, the folding top anti-folding end lifting method was used, in which the thumbs of both hands were placed on the dorsal distal fracture end, and the remaining four fingers held it and displaced the proximal end of the fracture to the palmar side, slowly increasing the palmar side into the angle and pressing the proximal end from the dorsal side with both thumbs. The palmar side into the angle should be increasing with the severity of the patient's shortening displacement and differentiation by palpating the injured area. When the dorsal cortical tops of the two fracture ends were palpated, both thumbs continuously pressed the dorsal distal fracture block to the palmar side, while the four fingers held the proximal end of the palmar displaced fracture to the dorsal side, with quick movements to reset it. During the resetting process, priority should be given to resetting the radius and then the ulna. (2) Splint fixation: four pieces of cedar bark were used for splinting, one flat pad was placed on the proximal palmar side, and one on the distal dorsal side of the fracture, and cotton gauze pads of moderate thickness were used to separate the splint from the skin. The proximal end of the splint length should be approximately over the upper one-third of the anterior wall, the distal dorsal plate required over the wrist for fixation, the distal ulnar plate and the distal radial plate to the radial styloid process for shaping the post and then placed at the base of the fifth metacarpal, fixed with a tie, and fixed in a neutral position on the anterior wall using a rotating middle plate, keeping the elbow flexed at 90° and suspended with a triangular scarf for 6 weeks. Weeks 1–2: Lifted the affected limb, applied local ice, and closely observed the blood circulation at the end of the affected limb; instructed the child to lightly flex and extend the interphalangeal joints, and practiced finger-splitting and finger-combining movements. Weeks 3–6: The children were instructed to increase finger flexion and extension mobility and to practice fist clenching, hand lifting, and shoulder shrugging. The child's family was instructed to bring the child to the hospital for regular review and to practice wrist flexion and extension, rotation, and other activities according to the healing condition of the fracture (Figure 1).

Control group: The surgery was performed under brachial plexus block anesthesia or general anesthesia with the child in a flat position and the affected limb in abduction. Radial surgery was performed first, and the incisions were all made through a longitudinal incision on the dorsal side of the radius, entered through the extensor tendon gap, and the fracture was repositioned and fixed with a T-shaped microplate. Ulna: A small incision was made on the dorsal side of the ulna with access between the extensor and flexor muscles to reset the fracture, and a flexible intramedullary nail (2 mm diameter) was driven into the proximal end of the ulna for internal fixation. Postoperatively, external fixation in plaster was performed. Antibiotics were routinely applied for about 5 days. The incision was changed once every 3 days, and attention was paid to adjust the elasticity of the external fixation of the cast.

All children were followed up at 4, 6, and 8 weeks after discharge, and the time to remove the keratoconus needle from the body was decided according to the review x-ray, while the splint or brace was removed and the affected limb was gradually exercised functionally. For the first 12 months after the surgery, outpatient review was conducted every 3 months; after that, outpatient review was conducted every 6 months.

### 2.6. Observation index

Efficacy evaluation: The fracture healing time of the two groups was recorded and compared. Clinical efficacy was evaluated using Anderson's criteria for forearm fracture (12): excellent—fracture healing, loss of elbow or wrist flexion and extension <10%, loss of forearm rotation <25%; good—fracture healing, loss of elbow or wrist flexion and extension <20%, loss of forearm rotation <50%; general—fracture healing, loss of elbow or wrist flexion and extension >30%, loss of forearm rotation >50%; poor—malunion or non-union of the fracture and loss of forearm motor function. Pain level: The visual analog scale (VAS) was used to assess the children's pain before treatment, at 3 weeks after treatment and at 7 weeks after treatment, with a total score of 0–10, the lower the score, the less severe the pain level.

### 2.7. Statistical analyses

SPSS 20.0 statistical software was used for analysis. The measurement data were expressed as mean ± standard deviation, and the independent sample *t*-test was used for comparison between groups; the count data were expressed as number (%), and the chi-square test or Mann–Whitney test was used for comparison between groups. Values of *P* < 0.05 were considered statistically significant. GraphPad Prism 8.0 was used to plot the statistical graphs.

## 3. Results

### 3.1. Comparison of general data of children in two groups

In this study, the children were divided into 41 cases in the observation group (closed manipulation combined with splint external fixation treatment) and 41 cases in the control group (incisional repositioning elastic nail combined with plate internal fixation). There was no statistical difference between the two groups in terms of gender and age, injury side distribution, and distribution of causes of injury by chi-square test (*P *> 0.05), and there was no statistical difference between the two groups in terms of time to visit the clinic after injury by *t*-test (*P *> 0.05), which was comparable (Table l).

**Table 1:** Comparison of general information of children in two groups.

Data		Observation group (*n* = 41)	Control group (*n* = 41)	*χ*^2^/*t*-value	*P*-value
Gender	Male	34 (82.93)	32 (78.05)	0.311	0.577
	Female	7 (17.07)	9 (21.95)		
Age	5–8 years	11 (26.83)	12 (29.27)	0.546	0.761
	9–11 years	13 (31.71)	10 (24.39)		
	12–14 years	17 (41.46)	19 (46.34)		
Side of injury	Left side	24 (58.54)	22 (53.66)	0.198	0.656
	Right side	17 (41.46)	19 (46.34)		
Cause of injury	Falling from a height	8 (19.51)	5 (12.20)	0.837	0.841
	Bicycle fall	10 (24.39)	11 (43.90)		
	Fall on flat ground	7 (17.07)	8 (19.51)		
	Traffic accident	16 (39.02)	17 (41.46)		
Time to visit after injury (h)		12.23 ± 8.06	12.52 ± 7.59	0.168	0.867

### 3.2. Comparison of clinical efficacy between two groups of children

The efficacy was evaluated according to the Anderson forearm fracture efficacy evaluation criteria, and the results of statistical analysis showed that there was no statistically significant difference between the efficacy of the two groups (*P *> 0.05) (Table 2).

**Table 2:** Comparison of clinical outcomes between the two groups of children.

Group	Excellent	Good	General	Poor	Excellent rate
Observation group (*n* = 41)	16 (39.02)	20 (48.78)	4 (9.76)	1 (2.44)	36 (87.80)
Control group (*n* = 41)	15 (36.59)	18 (43.90)	6 (14.63)	2 (4.88)	33 (80.49)
χ^2^/*z*-value	−0.570				0.823
*P*-value	0.569				0.364

### 3.3. Comparison of VAS scores of children in the two groups at different times

**Figure 2:**
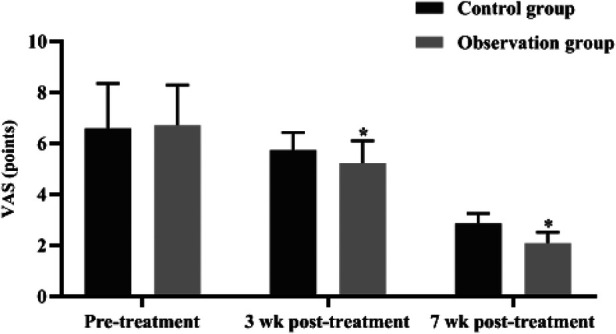
Comparison of VAS scores of children in the two groups at different times. *P < 0.05 compared with the control group in the same period.

At 3 and 7 weeks after treatment, the VAS scores of children in both groups decreased (*P *< 0.05), and the VAS scores of the observation group were significantly lower than those of the control group (*P *< 0.05), indicating that the observation group had a significant advantage in the relief of pain after treatment (Figure 2).

### 3.4. Comparison of fracture healing time between two groups of children

The fractures in both groups healed after treatment with two different methods. There was no statistically significant difference in the healing time of fractures between the two groups (*P* > 0.05). The observation group had significantly lower hospitalization time, treatment cost, and complication ratio than the control group (*P* < 0.05) (Table 3).

**Table 3:** Comparison of clinical observation indexes between two groups of children.

Group	Time to fracture healing (weeks)	Length of stay (d)	Treatment cost (Yuan)	Complications (%)
Observation group (*n* = 41)	5.23 ± 1.12	5.24 ± 2.43	4123.77 ± 702.56	7 (17.07)
Control group (*n* = 41)	5.14 ± 1.24	8.57 ± 2.72	6614.09 ± 622.36	16 (39.02)
t/χ^2^-value	0.345	5.846	16.989	4.895
*P*-value	0.731	0.000	0.000	0.027

## 4. Discussion

Displaced fractures of the distal radius and ulna in children are one of the serious fractures that often occur during sports in children, mostly closed fractures, and improper early management can easily lead to a series of sequelae, such as deformity of the injured arm and forearm dysfunction (13). Although, with the continuous improvement of modern treatment techniques, the incidence of injured arm deformity and forearm dysfunction has been significantly reduced, incomplete and suboptimal recovery of elbow joint function still exists, which is closely related to early management techniques, fixation methods, and functional training (14, 15). The purpose of anterior wall fracture repositioning is to restore its rotational function, and poor fracture repositioning may lead to limited joint motion and pain thereby triggering forearm fascial area syndrome (16); therefore, repositioning should be as close to anatomic alignment or achieve anatomic alignment as possible.

In recent years, incisional repositioning elastic nail combined with plate internal fixation has gradually become the most common method of internal fixation for the treatment of double fractures of the distal radial and ulnar segments in children. However, children often refuse to move because of the painful postoperative wound stimulation, which, together with prolonged braking of the affected elbow, leads to stagnation of blood stasis in the affected limb, blockage of qi and blood, loss of nourishment of tendons and bones, joint contracture and adhesions, and various combined factors that eventually lead to stiffness and limited movement of the elbow joint (17, 18). Manual repositioning has the characteristics of being less painful and less likely to cause joint damage, the time spent with external fixation after good repositioning is shorter, and the child's forearm function recovers quickly, which is one of the traditional treatment methods commonly used in clinical practice (19). It was concluded that patients with extremity fractures should be treated with manual repositioning, especially for patients with upper extremity fractures that do not damage the joint and are not severely separated, and that manual repositioning can significantly reduce injury and help fracture healing (20).

In this study, children in the control group were treated with incisional repositioning elastic nail combined with plate internal fixation and children in the observation group were treated with closed manipulation combined with splint external fixation. However, the incidence of complications was significantly higher in the control group than in the observation group, and the length of hospital stay was longer and the treatment cost was higher in the control group than in the observation group. It is suggested that after treatment with incisional repositioning elastic nail combined with plate internal fixation, there is some local damage to soft tissues due to the need for small intraoperative incisional repositioning, which can have some impact on the wound in the short term and require a longer time to recover, indirectly leading to a higher cost of treatment required than closed repositioning combined with splint external fixation (21, 22). However, the fracture healing time and preoperative and postoperative pain levels were comparable between the two groups of children, which may be related to the rich periosteum, abundant blood flow, and rapid healing in children. In addition, the efficacy was evaluated according to the Anderson forearm fracture efficacy evaluation criteria, and excellent rates of 87.80% and 80.49% were observed in the observation group and the control group, respectively; the difference between the two groups was not significant. This suggests that in children with displaced fractures of the distal radius and ulna, the treatment with closed revision manipulation combined with splint external fixation is comparable to the treatment with incisional repositioning elastic nail combined with plate internal fixation with a good rate of more than 80%, both of which can achieve better treatment results.

Therefore, the author believes that in children with displaced lower radial–ulnar fractures, the indications for surgery should not be expanded simply for anatomic or near-anatomic repositioning, or the treatment of displaced lower radial–ulnar fractures in children with adult standards increases the risk of treatment. Instead, a convenient and effective method should be chosen according to the patient's degree of injury, the physiological characteristics of the child, the wishes of the patient and the parents, and the strengths of osteopathic manipulation and splinting. Manual repositioning with splint external fixation can achieve satisfactory results in the treatment of displaced fractures of the distal radial and ulnar segment in children, and at the same time, the operation is simple with low cost, short hospitalization time, and few complications, which is especially suitable for promotion in primary hospitals and has good social benefits.

## 5. Typical case study

**Figure 3:**
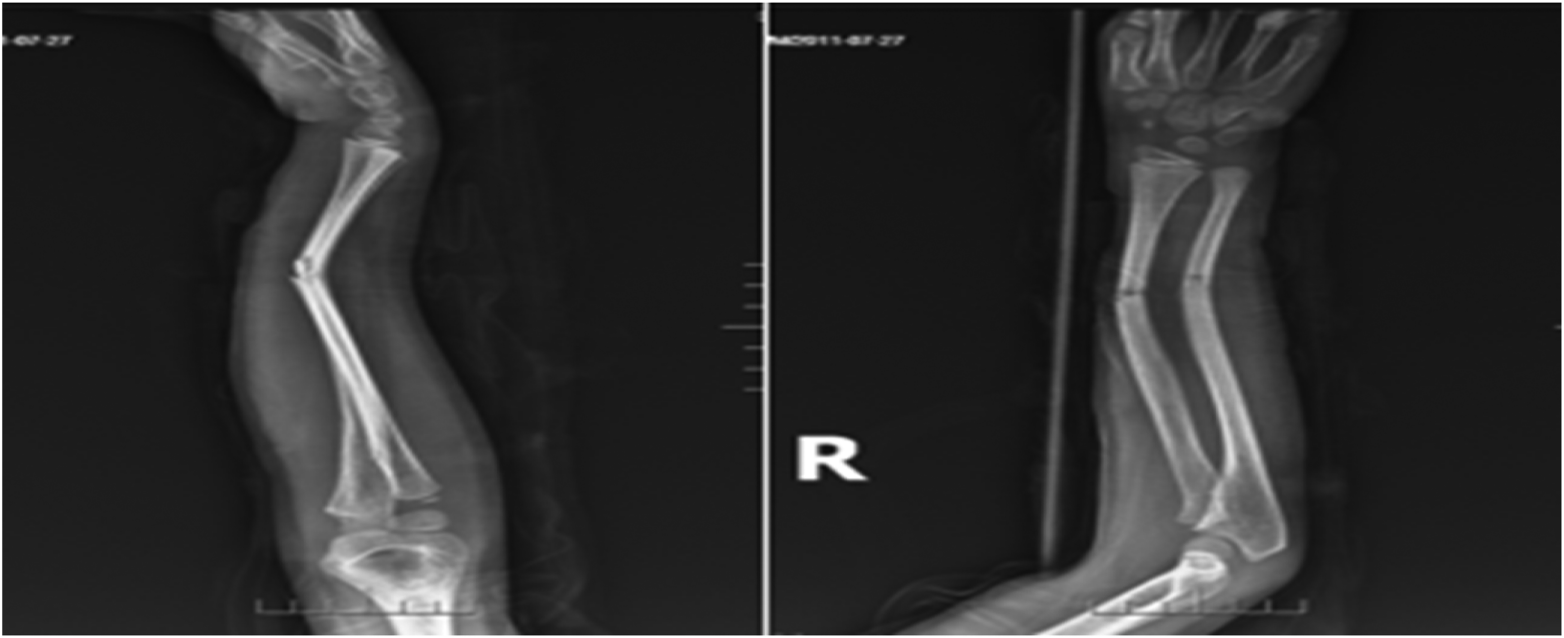
X-rays at the time of admission.

**Figure 4:**
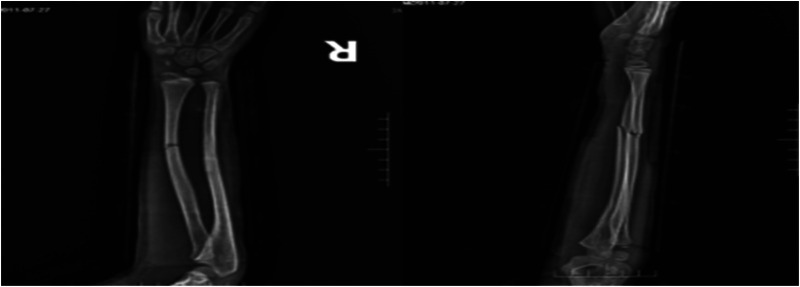
X-rays after manipulation.

**Figure 5:**
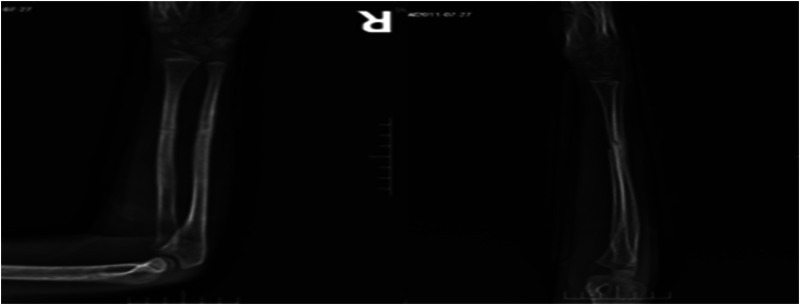
X-rays after 2 months of reset.

**Figure 6:**
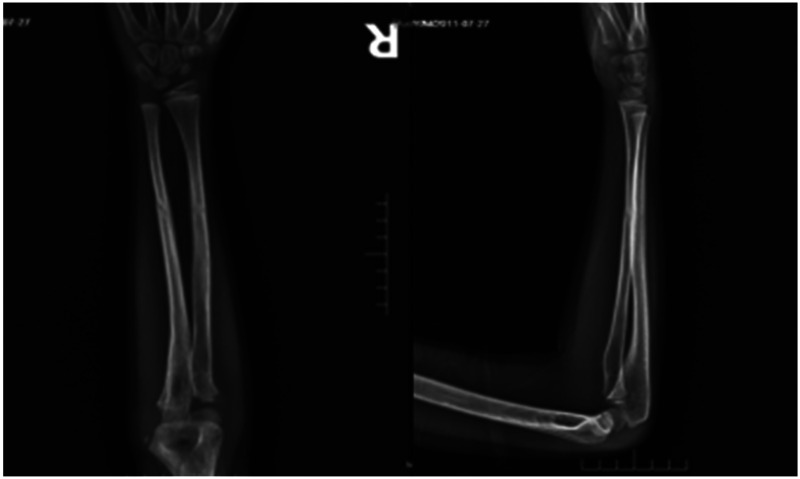
X-rays after 5 months of reset.

Case 1: A male child, 10 years old, fell while playing with a scooter and was admitted to the hospital with 4 h of swelling, deformity, and limitation of movement of the right forearm. On admission: deformity and swelling of the right forearm were obvious, with sharp pressure pain in the middle and lower ulnar radius, longitudinal percussion pain, localized palpable bone rubbing sensation, and abnormal activity. The right elbow joint was movable and the right wrist joint was limited. The radial artery pulsation was palpable and there was no abnormality in blood flow, sensation, and movement of the finger ends. At the time of admission, x-ray showed a fracture of the right radius and lower middle ulna, with the distal fracture end displaced to the north and the fracture end angled to the palmar side (Figure 3). The fracture was diagnosed as a fracture of the middle and lower segments of the right radius and ulna, and after excluding contraindications, the fracture was treated with manual repositioning combined with splint external fixation, and the x-ray showed good alignment of the fracture end after manual repositioning (Figure 4). The child was discharged from the hospital and followed up regularly. X-rays taken 2 months after the repositioning showed good alignment of the fracture end and local bone scab growth (Figure 5). X-rays taken 5 months after resetting showed that the fracture line had largely disappeared and met the criteria for bony healing of the fracture (Figure 6).

**Figure 7:**
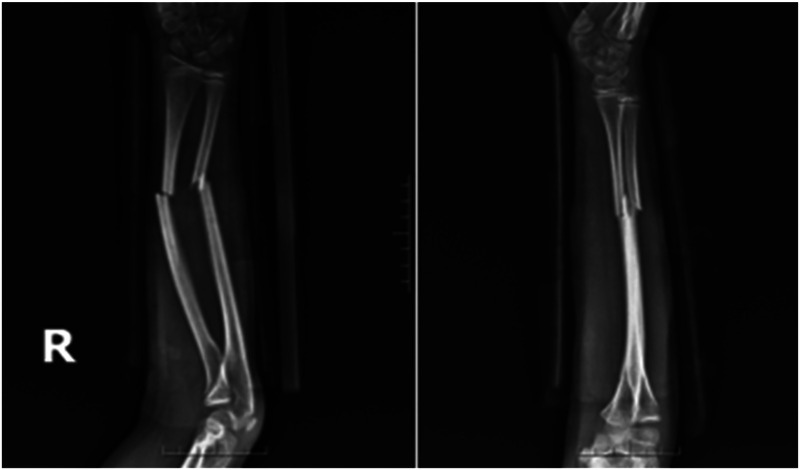
Preoperative x-rays.

**Figure 8:**
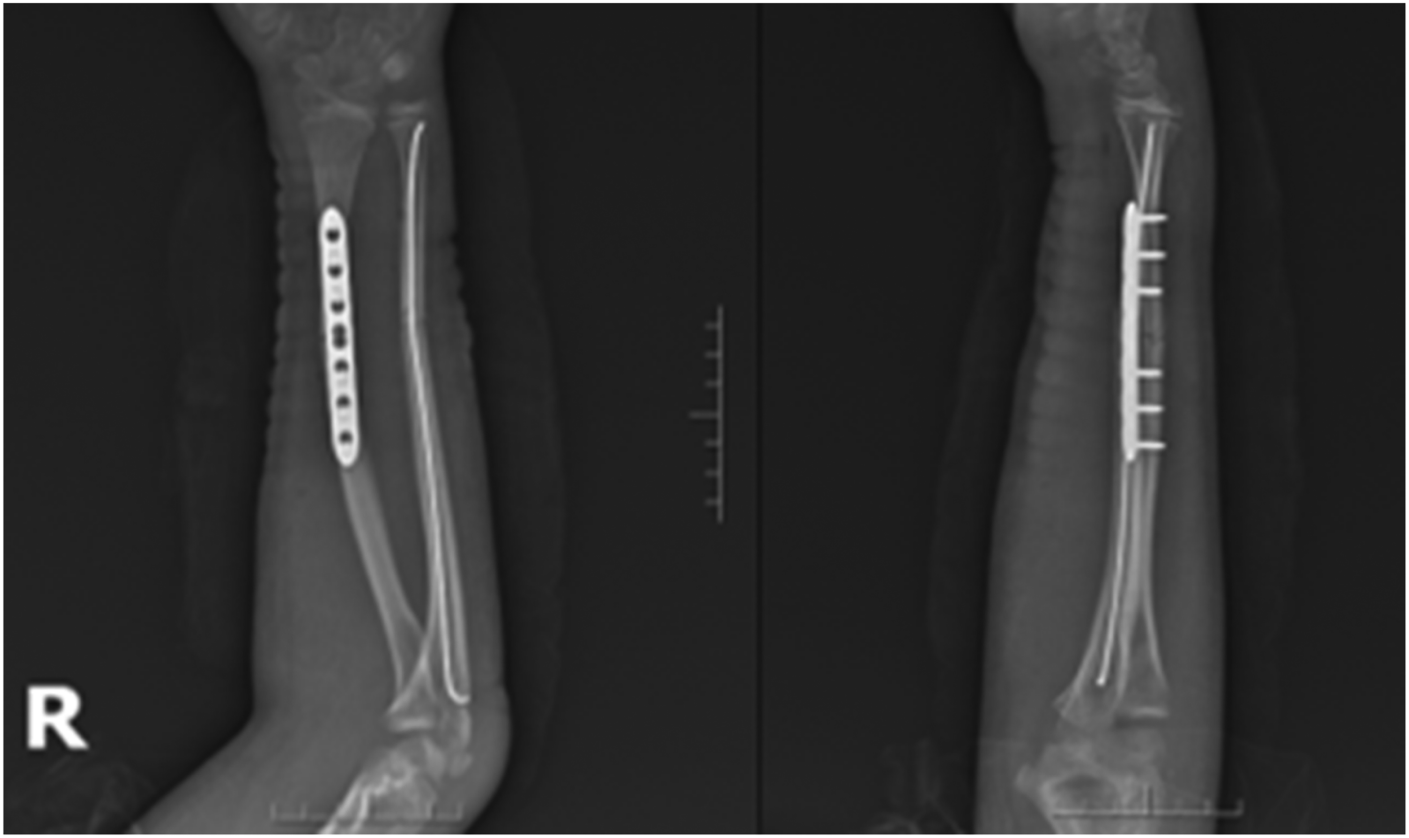
Postoperative x-rays.

**Figure 9:**
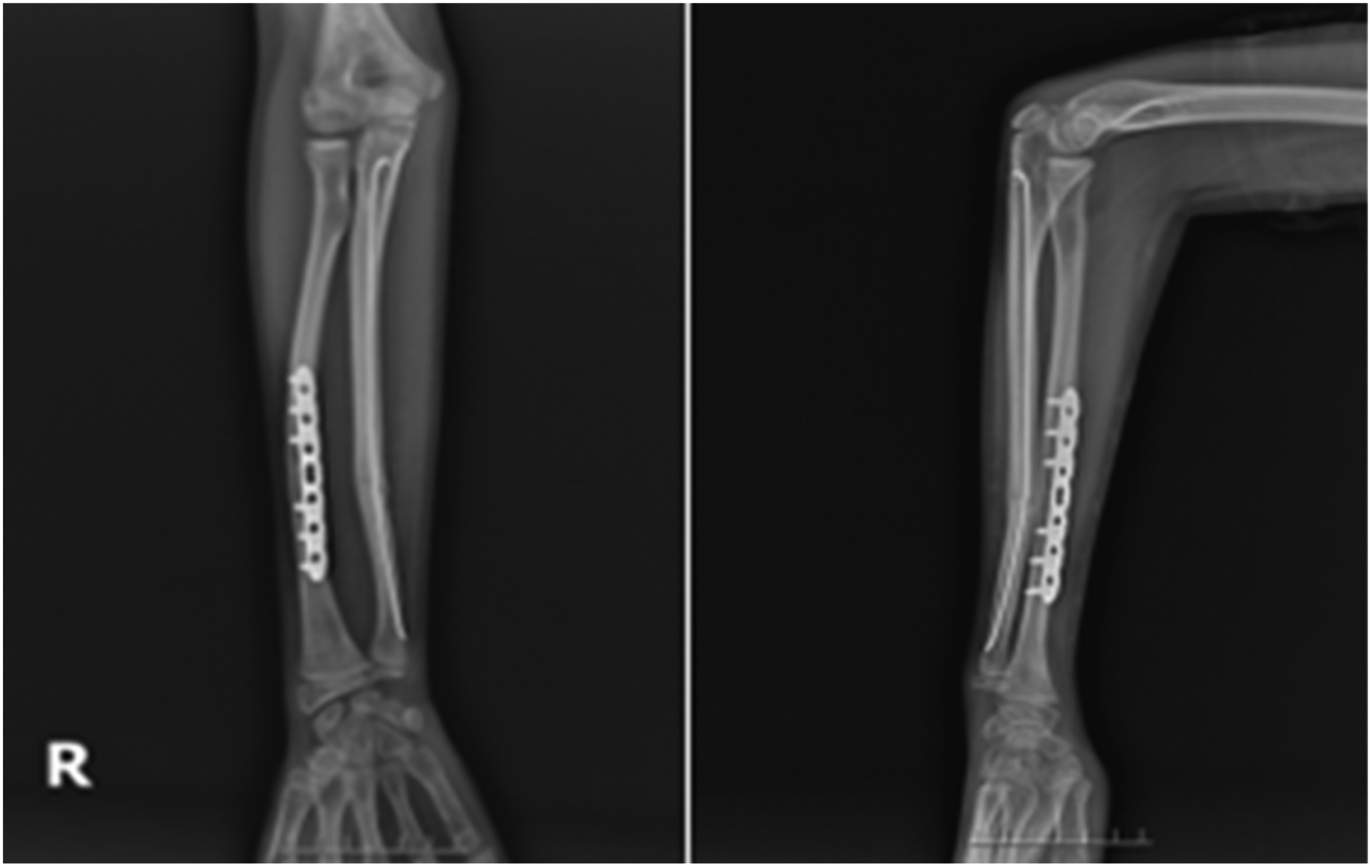
Postoperative x-ray at 5 months.

**Figure 10:**
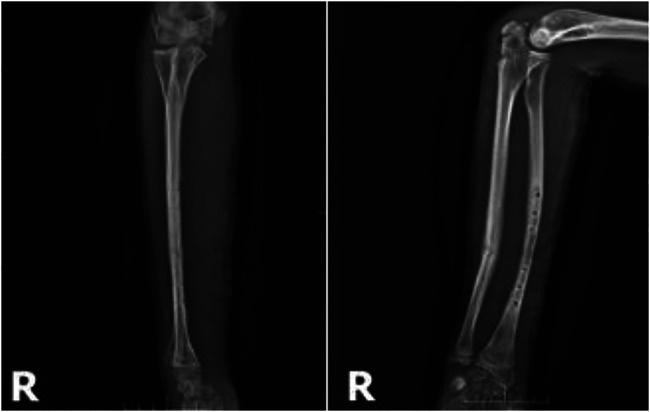
X-rays after removal of internal fixation at 5 months postoperatively.

Case 2: A male child, 12 years old, fell flat on the ground while playing and was referred to our hospital from an outside hospital with 6 h of swelling, pain, deformity, and limitation of movement in his right forearm. He was admitted to the hospital for examination: swelling and deformity of the right forearm were obvious, with sharp pressure pain in the middle and lower ulnar radius, longitudinal percussion pain, localized palpable bone rubbing sensation, and abnormal activity. The right elbow joint was movable, the right wrist joint was limited, radial artery pulsation was palpable, and there was no abnormality in blood flow, sensation, and movement of the finger ends. Preoperative radiographs suggested a fracture of the middle and distal radius and ulna, with the radial fracture end angled to the radial side and the distal fracture end displaced to the palmar and ulnar sides; the ulnar fracture end angled to the radial side and the distal fracture end displaced to the dorsal and ulnar sides (Figure 7). After excluding contraindications to surgery, the fracture was fixed with an incisional plate with elastic nail under brachial plexus anesthesia and external fixation in plaster after surgery. Postoperative x-rays showed good alignment of the fracture end (Figure 8). The child was discharged from the hospital and was followed up regularly. Radiographs taken at 5 months after surgery showed good healing of the fracture end and growth of bone scabs, which met the criteria for bony healing (Figure 9). The internal fixation was removed and x-rays were taken to observe the healing of the fracture, which showed good healing of the fracture end (Figure 10).

## Data Availability

The original contributions presented in the study are included in the article/Supplementary Material, further inquiries can be directed to the corresponding author.
